# Growth Monitoring: A Survey of Current Practices of Primary Care Paediatricians in Europe

**DOI:** 10.1371/journal.pone.0070871

**Published:** 2013-08-05

**Authors:** Pauline Scherdel, Jean-François Salaün, Marie-Noëlle Robberecht-Riquet, Laura Reali, Gabriella Páll, Elke Jäger-Roman, Manuel Praena Crespo, Marilena Moretto, Margareta Seher-Zupančič, Sigurlaug Agustsson, the European Confederation of Primary Care Paediatricians (ECPCP) research group, Martin Chalumeau

**Affiliations:** 1 Institut National de la Santé et de la Recherche Médicale, Centre for research in Epidemiology and Population Health, U1018, Lifelong Epidemiology of Diabetes, Obesity and Kidney Diseases Team, Villejuif, France; 2 Univ Paris-Sud, UMRS 1018, Villejuif, France; 3 Pediatric office, St-Brieuc, France; 4 Association Française de Pédiatrie Ambulatoire, Gradignan, France; 5 Pediatric office, Mons-en-Baroeul, France; 6 Associazione Culturale Pediatri Cultural Association of Pediatrician, Roma, Italy; 7 National Institute of Child Health, Child Health Information and Research Department, Budapest, Hungary; 8 Berufsverband der Kinder-und Jugendärzte, Köln, Germany; 9 Health Center La Candelaria, University of Seville, Seville, Spain; 10 Paediatric Department, Hôpital Saint Pierre, Free University of Brussels, Brussels, Belgium; 11 Zdravstveni dom Velenje, Velenje, Slovenia; 12 Centre of Pediatrics duVal Sainte Croix, Luxembourg, Luxembourg; 13 European Confederation of Primary Care Paediatricians, Lyon, France; 14 Department of Pediatrics, Necker-Enfants Malades Hospital, Paris Descartes University, Paris, France; 15 Assistance Publique Hôpitaux de Paris, Paris, France; 16 Institut National de la Santé et de la Recherche Médicale, U953 Epidemiological Research Unit on Perinatal Health and Women's and Children's Health, Paris, France; Hospital for Sick Children, Canada

## Abstract

**Objective:**

We aimed to study current practices in growth monitoring by European primary care paediatricians and to explore their perceived needs in this field.

**Methods:**

We developed a cross-sectional, anonymous on-line survey and contacted primary care paediatricians listed in national directories in the 18 European countries with a confederation of primary care paediatricians. Paediatricians participated in the survey between April and September 2011.

**Results:**

Of the 1,198 paediatricians from 11 European countries (response rate 13%) who participated, 29% used the 2006 World Health Organization Multicentre Growth Reference Study growth charts, 69% used national growth charts; 61% used software to draw growth charts and 79% did not use a formal algorithm to detect abnormal growth on growth charts. Among the 21% of paediatricians who used algorithms, many used non-algorithmic simple thresholds for height and weight and none used the algorithms published in the international literature. In all, 69% of paediatricians declared that a validated algorithm to monitor growth would be useful in daily practice. We found important between-country variations.

**Conclusion:**

The varied growth-monitoring practices declared by primary care paediatricians reveals the need for standardization and evidence-based algorithms to define abnormal growth and the development of software that would use such algorithms.

## Introduction

Growth monitoring can be summarized in a five-point paradigm first described by Garner: 1) health professionals regularly measure the height and weight of children; 2) they plot the information on a growth chart; 3) when growth is abnormal, they start appropriate investigations; 4) as a result, a serious condition is diagnosed earlier; and 5) the prognosis is improved by the earlier diagnosis [1]. This simple paradigm is accepted worldwide but raises many questions: Which growth charts should be used? [international charts such as from the World Health Organization (**WHO**) Multicentre Growth Reference Study (**WHO-MGRS**) [2] or national ones such as from the US Centers for Disease Control and Prevention–National Center for Health Statistics (**CDC-NCHS**), Hesse, Fundación Faustino Orbegozo etc.] [3]–[6]; Which diseases should be targeted? [7]–[9]; and How should abnormal growth be defined? (with simple criteria [8], [10] or complex algorithms [11]–[13]). Moreover, a weakness of most growth charts is the absence of longitudinal information. Because of their cross-sectional nature, growth charts provide a snapshot of population growth at only one time, with no information about centiles crossing over time [14], [15]. Furthermore, no reliable evidence exists to support the effectiveness of this worldwide monitoring of growth [1], but empirical evidence supports a high prevalence of diagnosis delays [7], [16]–[18] and inappropriate referrals [19].

Growth-monitoring performance depends on growth-monitoring practices, mainly the type of growth charts and the referral criteria used [7], [20]. Growth charts are considered an essential clinical tool for monitoring growth disorders [1] and are widely used [6]. Recently, new growth charts from the WHO [2] show curves for healthy children under optimal conditions, which has raised questions about the type of growth charts to use for evaluating growth in children. However, data on growth-monitoring practices show important variations within [6] and between countries in terms of the type of growth charts used [3] and the choice of auxological referral criteria [13], [16]. But, current available data on growth-monitoring practices are limited to a small survey among hospital-based pediatric endocrinologists [6] and studies performed in the 1990s [7]. The introduction of the WHO growth charts, the recent availability of growth-monitoring software and the relatively recent publication of evidence-based algorithms [12], [13], [17], [19], [21]–[23] may have modified these practices.

We aimed to study the current practices in growth monitoring among European primary care paediatricians and to explore their perceived needs in this field.

## Methods

We developed a European, cross-sectional, anonymous, on-line survey of growth-monitoring practices (Appendix S1). Participants were eligible to answer the survey if they practiced primary care pediatrics in one of the 18 countries containing a European Confederation of Primary Care Paediatricians (**ECPCP**), a medical society of primary care paediatricians in Europe established to exchange scientific information and improve professional practice [24]. The study was approved by the ECPCP research group and the Institutional Review Committee (Comité de Protection des Personnes Ile de France III) stated that ‘‘this research was found to conform to generally accepted scientific principles and research ethical standards’’. Primary care paediatricians in national directories of the 18 ECPCP countries were contacted to participate in the survey between April and September 2011. Given the exploratory nature of the study, we chose an arbitrary number of at least 10 participants in each country to participate and did not aim to obtain a representative sample of the population of primary care paediatricians.

The on-line survey comprised 11 items asking about paediatrician characteristics and growth-monitoring practices, specifically country of origin and type of practice, growth charts used (local, national, international), tools used for detecting growth abnormalities (height velocity, algorithms etc.) and perceived needs for software and algorithms for detecting growth abnormalities. This questionnaire was validated on 15 primary care peadiatricians with a preliminary survey performed in 2010 from 10 members countries of ECPCP.

Growth-monitoring practices of responding paediatricians were described. Then, we used a two-level hierarchical logistic regression model with paediatricians (level 1) nested within countries (level 2) for studying whether the specialization declared by paediatricians explained the variations in growth-monitoring practices. A different model was constructed for each growth monitoring practices (WHO-MGRS curves, an algorithm to detect abnormal growth, and software to monitor growth). First, we estimated a random intercept model without any variable to obtain the baseline country-level variance (σ^2^1), and we assessed variations in practices across countries. In a second model, we included the specialization declared by paediatricians and estimated the country-level variance (σ^2^2) after adjustment for this paediatrician-level variable. We used the proportional change in the variance (PCV) defined as PCV = [(σ^2^1)–(σ^2^2)]/(σ^2^1) to assess the extent to which country differences may be explained by the specialization of paediatricians. Descriptive analyses and creation of multi-level models involved use of SAS v9.3 (SAS Inst., Cary, NC).

## Results

Among the 16 ECPCP countries who participated, we *a posteriori* excluded 5 (Czech Republic, Latvia, Lithuania, Slovakia, and Sweden) because of fewer than 10 respondents from each country. Thus, the results are based on the answers for 1,198 participants from 11 ECPCP countries (response rate 13% - Table 1). Most of the respondents were from France (42%), Spain (20%), Germany (14%), and Italy (8%). In all, 27% of respondents declared a specialization (including 5% in gastroenterology and nutrition, 4% in endrocrinology), with significant differences among countries (from 61% in Hungary to 13% in Belgium, p<0.001). A total of 69%, 29%, and 2% of respondents used national, WHO-MGRS and CDC-NCHS growth charts, respectively, with significant between-country variations (WHO-MGRS charts: from 51% in Italy to 17% in Luxemburg, p<0.001). Among respondents, 61% declared using software to monitor growth (from 85% in Italy to 0% in Portugal and Slovenia, p<0.001). In all, 21% of responding paediatricians declared using an algorithm to detect abnormal growth, with significant between-country variation (from 58% in Hungary to 0% in Switzerland, p<0.001). In all cases, this algorithm involved simple thresholds for height, weight, body mass index or height velocity, and no respondents declared using any of the algorithms published in the international literature [8], [12], [17]. Among pediatricians who did not declare using an algorithm, 69% indicated that it would be useful in their daily practice (from 100% in Belgium to 57% in Switzerland, p = 0.001). Variations in growth-monitoring practices were poorly explained by the specialization declared by paediatricians, which accounted for 13.8%, 1.5% and 0.0% of the between-country variations in the use of WHO-MGRS curves, an algorithm to detect abnormal growth and software to monitor growth, respectively.

**Table 1:** Primary care paediatricians and their growth-monitoring practices by country (N = 1198).

Country of origin	No. of PCPsin country	No. of PCPsin theECPCP*	Theoretical no. of paediatricians receiving the survey	No. of respondents	No. of paediatricians with a specialization (n = 1,198)	Use of WHO-MGRS growth chart (n = 1,065)	Hierarchy in use of WHO-MGRS growth charts	Use of an algorithmto detect abnormalgrowth (n = 673)	Use of softwareto monitorgrowth (n = 747)	Main growth chart(s) used (reference)
				p**<0.001	p**<0.001	p**<0.001		p**<0.001	p**<0.001	
Belgium	NC	NC	150	15 (10.0)	2 (13.3)	3 (20.0)	2^nd^	1 (8.3)	5 (41.7)	Flemish Growth Charts [35]
France	2550	1600	1500	506 (33.7)	120 (23.7)	85 (19.4)	2^nd^	56 (22.8)	179 (64.4)	“Sempé” [36]
Germany	6541	5661	2500	172 (6.9)	43 (25.0)	41 (25.2)	2^nd^	19 (17.6)	91 (79.1)	“Hesse” [37]
Hungary	1586	1260	830	67 (8.1)	41 (61.2)	27 (47.4)	2^nd^	19 (57.6)	20 (50.0)	“Joubert” [38]
Israel	900	700	NC	22 (NC)	8 (36.4)	10 (47.6)	2^nd^	2 (14.3)	14 (82.4)	CDC-NCHS [39]
Italy	7000	1000	1200	100 (8.3)	35 (35.0)	45 (50.6)	3^rd^	13 (21.7)	56 (84.9)	Tanner [40] or CDC-NCHS [39]
Luxemburg	NC	NC	60	13 (21.7)	2 (15.4)	2 (16.7)	3^rd^	1 (11.1)	1 (11.1)	Luxemburgish Growth chartsor “Prader” [41]
Portugal	NC	80	NC	11 (NC)	4 (36.4)	5 (50.0)	1^st^	1 (12.5)	0 (0.0)	WHO-MGRS [39]
Slovenia	190	170	90	22 (24.4)	4 (18.2)	4 (20.0)	3^rd^	4 (30.8)	0 (0.0)	CDC-NCHS [39] or Miscellanous
Spain	6424	5224	2490	239 (9.6)	52 (21.8)	82 (39.1)	2^nd^	23 (15.8)	92 (56.1)	“Hernández-Fundación Faustino Orbegozo” [42]
Switzerland	1216	747	NC	31 (NC)	8 (25.8)	9 (30.0)	2^nd^	0 (0.0)	1 (4.0)	“Prader” [41]
Total				1,198 (12.9)	319 (26.6)	313 (29.4)	2 (median)	139 (20.7)	459 (61.4)	

Data are number (%).

PCP, primary care physicians; WHO-MGRS, World Health Organization Multicentre Growth Reference Study; CDC-NCHS, US Centers for Disease Control and Prevention–National Center for Health Statistics; NC, Not communicated.

* as reported on the European Confederation of Primary Care Paediatricians website [24].

** comparison between countries.

## Discussion

Our results show important between-country differences in growth-monitoring practices among 1,198 primary care paediatricians from 11 European countries. Almost one-third declared using the 2006 WHO-MGRS charts to monitor the growth of children, almost two-thirds used software to analyze growth, and one-fifth used an algorithm to detect abnormal growth. Cited algorithms were all non-algorithmic simple thresholds for height, weight, body mass index or height velocity. None of the respondents declared using any of the 5 algorithms published in the international literature [8], [10]–[13].

In developed countries, where the main purpose for monitoring the growth of healthy children is mass screening to enable early diagnosis of serious conditions, the availability of the 2006 WHO-MGRS may have an important impact on growth-monitoring practices. These new growth charts have been adopted by peadiatricians in many countries and were used by about 30% of our peadiatricians and were the second most-used growth charts [25]. The use of these new growth charts (versus national charts) may modify the interpretation of growth, a key step in growth-monitoring practices [4]. The adoption of the 2006 WHO-MGRS growth charts for growth monitoring at a national level is encouraged by the WHO, but epidemiologic and clinical consequences of such adoption are being evaluated at national levels. Differences have been found between national curves and WHO-MGRS curves in many countries [20], [26]–[28]. For example, 3-year-old Hong Kong children are smaller than WHO children at that age [29] whereas Belgian and Norwegian children up to 2 years old are taller than WHO children at that age [30]. Furthermore, for children older than 5 years, the 2006 WHO-MGRS growth charts were based on growth data previously used for creating the US CDC-NCHS reference in 1977. The use of this curve, considering the secular trend for height [31], could lead to erroneous conclusions about abnormal growth. Standardization of growth charts is needed for defining normal growth and correctly applying algorithms.

Important next steps toward an evidence-based screening programme are the identification of target conditions and the definition of abnormal growth. Target conditions should have the following attributes: 1) a natural history including a long period when the main symptoms are auxological; and 2) a high level of evidence demonstrating that an early diagnosis is associated with a better outcome. The standardization of the definition of abnormal growth requires external validation and comparison of existing clinical decision rules [32], [33] and/or their refinement or the development of new ones. Currently, 5 algorithms have been published by Dutch and British teams, and the WHO. These algorithms involved a simple single threshold [8], [10] or complex combinations of auxological criteria [11]–[13]; four were derived by consensus among experts [8], [10], [11], [13] and one was a clinical decision rule derived with patients data. Their performance (sensitivity, specificity) [19], [34] and/or their levels of validation are low [12]. The low rate of use of these algorithms we observed (0%) is probably explained by the lack of information about the existence of these rules, their low performance and/or validation levels, and/or the complexity of some of them.

The present survey has several limitations. Our sample is not representative of all paediatricians from the 18 ECPCP countries because it involved volunteer paediatricians from 11 ECPCP countries. We removed data for five countries with an insufficient number of responding paediatricians. The variable response rate between countries led to an over-representation of paediatricians from France, Spain and Germany. Declaration bias is possible in on-line surveys because of the subjective declaration of growth-monitoring practices by paediatricians. Indeed, we relied on the declaration by paediatricians because we could not follow the paediatricians’ real day-to-day practices. However, our survey results confirm previous results, especially those from 2005 Grote et al. study, involving members of the European Society of Pediatric Endocrinology [3], [4]. Finally, neither the present survey nor previous ones targeted general practitioner practices despite the importance of these physicians in growth monitoring in many European countries.

In conclusion, this survey identified important opportunities to standardize practices to monitor growth of children, practices that are not currently evidence based and are not in accordance with screening standards [8]. This survey demonstrates the need for validated evidence-based algorithms to define abnormal growth (by validating existing algorithms or deriving new ones). The implementation of such algorithms through newly developed software seems possible, given that many of our respondents used software to monitor growth.

## Supporting Information

Appendix S1 **On-line survey.**
(DOCX)
