# Supplementary material for: Growth Monitoring: A Survey of Current Practices of Primary Care Paediatricians in Europe
Source: PLoS One. 2013 Aug 5;8(8):e70871. doi: 10.1371/journal.pone.0070871 (PMC3734305; doi:10.1371/journal.pone.0070871)
Supplement: Appendix S1 — On-line survey. (DOCX) [file pone.0070871.s001.docx]

**Appendix:**

**On-line survey**

Country where you work:

Superspecialization :

No

Yes endocrinology nutrition other, please precise: ________

Do you use the 2006 Multicenter Growth Reference Study World Health Organisation (MGRS/WHO) growth charts in daily practice to monitor growth in your patients?

Yes No

If you have answered no, could you please indicate which growth chart do you use in daily practice? (Please be as precise as possible for the name and the reference of this growth chart) ________

Do you use height velocity to monitor growth?

Yes No

Do you use software to automatically draw growth charts based on the patient’s height and weight data?

Yes No

If you have answered yes, please give us the exact references of this software: ________

Do you use a formalised algorithm to detect abnormal growth on growth charts?

Yes No

If you have answered yes, please indicate precisely which algorithm: ________

If you have answered no, do you think such an algorithm would be useful in your daily practice?

Yes No
